# Mosquitoes of Western Yunnan Province, China: Seasonal Abundance, Diversity, and Arbovirus Associations

**DOI:** 10.1371/journal.pone.0077017

**Published:** 2013-10-11

**Authors:** Hai-Lin Zhang, Yu-Zhen Zhang, Wei-Hong Yang, Yun Feng, Roger S. Nasci, Jie Yang, Yong-Hua Liu, Chao-Liang Dong, Shi Li, Bao-Sen Zhang, Zheng-Liu Yin, Pi-Yu Wang, Shi-Hong Fu, Ming-Hua Li, Fen Liu, Juan Zhang, Jie Sun, Can-Wei Li, Xiao-Yan Gao, Hong Liu, Huan-Yu Wang, Lyle R. Petersen, Guo-Dong Liang

**Affiliations:** 1 Yunnan Institute of Endemic Disease Control and Prevention/ Yunnan Provincial Center of Virus and Rickettsia Research, Dali, Yunnan, P.R. China; 2 Division of Vector-Borne Diseases, Center for Disease Control and Prevention, Fort Collins, Colorado, United States of America; 3 Dehong Prefecture Center for Disease Control and Prevention, Mangshi, Yunnan, P.R. China; 4 Ruili City Center for Disease Control and Prevention, Ruili, Yunnan, P.R. China; 5 Yunnan Institute of Parasitic Diseases, Simao, Yunnan, P.R. China; 6 State Key Laboratory for Infectious Disease Prevention and Control, Institute for Viral Disease Control and Prevention, Chinese Center for Disease Control and Prevention, Beijing, P.R. China; University of Texas at El Paso, United States of America

## Abstract

**Objective:**

The western borderland between Yunnan Province, China, and Myanmar is characterized by a climate that facilitates year-round production of mosquitoes. Numerous mosquito-transmitted viruses, including Japanese encephalitis virus circulate in this area. This project was to describe seasonal patterns in mosquito species abundance and arbovirus activity in the mosquito populations.

**Methods:**

Mosquitoes were collected in Mangshi and Ruili cities of Dehong Prefecture near the border of China and Burma in Yunnan Province, the Peoples Republic of China in 2010. We monitored mosquito species abundance for a 12-month period using ultraviolet light, carbon dioxide baited CDC light and gravid traps; and tested the captured mosquitoes for the presence of virus to evaluate mosquito-virus associations in rural/agricultural settings in the area.

**Results:**

A total of 43 species of mosquitoes from seven genera were collected, including 15 *Culex* species, 15 *Anopheles* spp., four *Aedes* spp., three *Armigeres* spp., one *Mimomyia* spp., two *Uranotaenia* spp. and three *Mansonia* spp.. Species richness and diversity varied between Mangshi and Ruili. *Culex tritaeniorhynchus*, *Culex quinquefasciatus*, *Anopheles sinensis* and *Anopheles peditaeniatus* were the most abundant species in both sampling sites. Ultraviolet light traps collected more specimens than CDC light traps baited with dry ice, though both collected the same variety of mosquito species. The CDC gravid trap was the most effective trap for capture of *Culex quinquefasciatus*, a species underrepresented in light trap collections. A total of 26 virus strains were isolated, which included 13 strains of Japanese encephalitis virus, four strains of Getah virus, one strain of Oya virus, one strain from the *orbivirus* genus, and seven strains of *Culex pipien pallens* densovirus.

**Conclusions:**

The present study illustrates the value of monitoring mosquito populations and mosquito-transmitted viruses year-round in areas where the climate supports year-round adult mosquito activity.

## Introduction

Yunnan Province is located in Southwest China, adjacent to Burma, Laos and Vietnam. Ecologically, Yunnan Province supports year-round production of mosquitoes and transmission of mosquito-borne viruses because of its low altitude and climate, which is borderline tropical - South sub-tropical, characterized by high temperature, humidity and rainfall during half of the year, and moderate temperature and rainfall conditions during the winter months. Human cases of Japanese encephalitis (JE) occur commonly in Yunnan Province [1] and Japanese encephalitis virus (JEV) (Genus Flavivirus, Family *Flaviviridae*), Dengue virus (DENV) (Genus Flavivirus, Family *Flaviviridae*), Chikungunya virus (CHIKV) (Genus *Alphavirus*, Family *Togaviridae*), Sindbis virus (SINV) (Genus *Alphavirus*, Family *Togaviridae*), Getah virus (GETV) (Genus *Alphavirus*, Family *Togaviridae*), Batai virus (BATV) (Genus *Orthobunyavirus*, Family *Bunyaviridae*), Banna virus (BAV) (Genus *Seadornavirus*, Family *Reoviridae*), Yunnan orbivirus (YUOV) (Genus *Obivirus*, Family *Reoviridae*) and several other viruses have been isolated from human patients and from mosquitoes collected in Yunnan Province [2-18]. Previous investigations describing mosquitoes and their association with arboviruses [3-8,10,14,16-21], have been limited to summer months and do not provide information about the ecology of vectors and viruses during periods when mosquito and virus activity is low. This information is critical for preventative vector-control interventions aimed at reducing virus amplification in subsequent seasons. 

The primary objective of this project is to describe 12-month seasonal patterns in mosquito species abundance and arbovirus transmission activity in western Yunnan Province and secondarily, evaluate the performance of three different mosquito collecting traps in sampling different mosquito species at different times of year. 

## Methods

### Ethics Statement

During mosquito collections, the local residents allowed us to access to their residences and land. 

### Collection Sites

**Figure 1:**
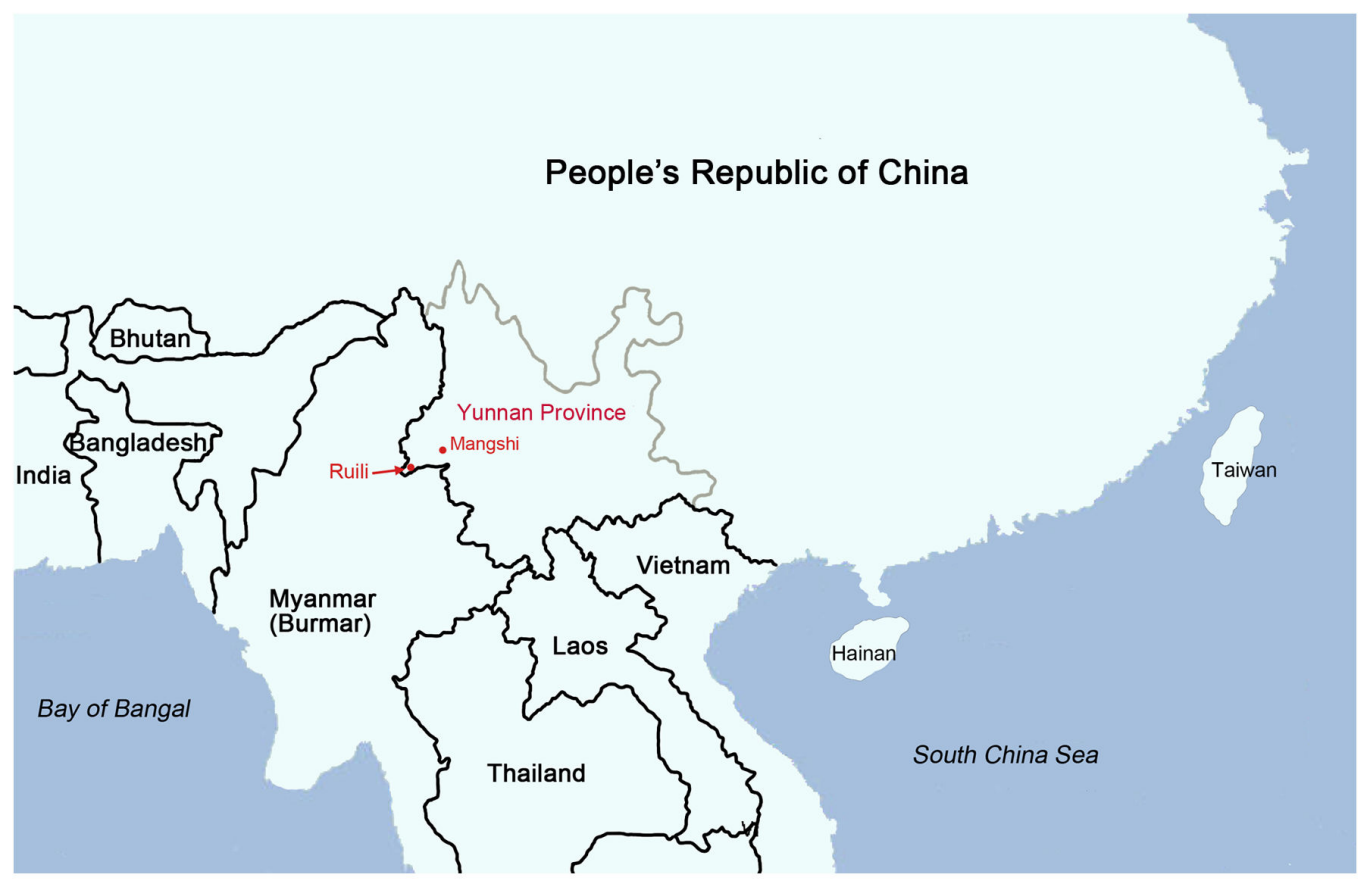
Location of villages in Mangshi and Ruili, Yunnan Province, China, where mosquito collection occurred.

Mosquitoes were collected in Yunnan Province, the Peoples Republic of China, in 2 villages each near the cities of Mangshi (previously known as Luxi city) and Ruili of Dehong Prefecture located near the Myanmar border (Table 1, Figure 1). The villages where collections were carried out are similar and characterized as agricultural with residential farms, supporting irrigated rice fields, row crops, and vegetable gardens, and scattered fish ponds. Pigpens were usually located adjacent to the residential houses. Weather data (monthly average temperature and relative humidity, and total precipitation) were obtained from weather stations at local airports located near each city. In general, Ruili, located approximately 80 km to the NW is warmer and wetter than Mangshi.

**Table 1:** Geographic location and elevation of villages where mosquito collections occurred from Jan-Dec 2010.

**City**	**Average Temp. °C**	**Average Rainfall**	**Villages/site**	**Longitude**	**Latitude**	**Elevation**
Mangshi	20.9/9.8 (high/low)	960 mm	Lameng/1	98.565175	24.39278	849 m
			Lameng/2	98.567766	24.39231	850 m
			Lahui/1	98.58739	24.47483	869 m
			Lahui/2	98.542197	24.47391	872 m
Ruili	27.4/15.8 (high/low)	1,454 mm	Hexing/1	97.80429	23.98538	729 m
			Hexing/2	97.81492	23.99796	718 m
			Qidui/1	97.88141	24.03757	740 m
			Qidui/2	97.88329	24.03808	729 m

### Mosquito Sampling Procedures

Two residences in each of the 4 rural villages, were selected as sampling sites. Each of the selected residences had pigs or cows on the property that were housed adjacent to farmland or wooded areas. At each site mosquito sampling was carried out with three types of mosquito traps. 

#### Traditional Trap (TT)

Kongfu Xiaoshuai trap (Wuhan Jixing Environmental Protection Scientific and Technological Limited-liability Company) is designed to collect host-seeking female mosquitoes and has been used extensively in previous surveys carried out in Yunnan Province [5-7,16]. The trap contains an ultraviolet (UV) light source and a fan, and utilizes 220V power source. For this study, this type of trap was placed in close proximity to each residence (often next to the pigpen) where a power supply was available. 

#### Light Trap (LT)

The CDC miniature light trap (BioQuip Products, Rancho Dominguez, CA, USA) also traps host-seeking mosquitoes, has a light source and uses dry ice as a carbon dioxide bait [22]. Because these traps are powered by D-cell batteries, they were placed in wooded areas adjacent to the houses. 

#### Gravid Trap (GT)

The CDC Gravid Trap [23] (BioQuip Products, Rancho Dominguez, CA, USA), was designed with a plastic basin (550 mm × 400 mm × 200 mm) in which a fresh mixture of pig manure and water was made each night to attract gravid mosquitoes. Gravid traps were placed within 15 m of pigpens or cow sheds near the residence. 

Mosquito collections were conducted for two consecutive nights each month, at each site, for a total of 16 trap nights/trap type/month. Traps were set at 1700-1900 h on each collecting day and mosquitoes were removed from the trap at 0700-0800 the following morning. Collected mosquitoes were immediately transported from the field sites to the laboratory and killed with cold by placing them at -20 °C for 30 min. Specimens were then identified to species and sex, and counted on a chill table. Following identification to species, females were sorted into pools of up to 100 specimens according to species, date, location, and trap type. Pools of mosquitoes were stored on liquid nitrogen until processed for arboviruses at the laboratory of the Yunnan Institute of Endemic Control and Prevention in Dali, Yunnan Province. 

### Identification of Mosquito Species and Estimation of Abundance, Species Richness and Diversity

Results were tabulated by species, sex, date, location, and trap type in order to evaluate efficiency of the different trap types and differences in mosquito community structure and relative abundance by location. Since trap effort was consistent among cities (i.e., the same number of traps of each type run each month), only two trap failures were recorded during the entire project, and additional trap days were added at the affected sites to compensate for the trap failures, overall measures of species abundance are given as the sum of mosquitoes of a given species from all three trap types during a given collecting month. 

Species richness is represented by the number of species collected at each location. Species diversity for each location was estimated using the Simpson Diversity Index (SDI), which is calculated as 1-(Σ n(n-1) / N(N-1)) where n=the total number of mosquitoes of a species and N = the total number of mosquitoes of all species in each location [24]. The SDI represents the probability that two randomly-selected individuals in a community are of different species. An index of 0 indicates no species diversity (i.e. each sampled member of the community belongs to a same species) whereas an index of 1 indicates maximum diversity. SDI gives more weight to the more abundant species and rare species only cause small changes in the value.

### Statistical Analysis

Due to the difference of latitude, altitude, humidity, and rainfall between Mangshi and Ruili, statistical analysis was conducted to compare species richness and the total number collected between these two sampling locations. Different trap types were also statistically compared for their abilities in colleting mosquitoes.

The t-Test was performed to compare SDI and species richness between Mangshi and Ruili.

The Z-Test was used to compare means of populations characterized by a Poisson distribution. In this study, Z-test was performed to compare mean number of specimens and number of species collected among different trap types, and to compare the abundance of the four most commonly collected species in Ruili and Mangshi. 

The Mann-Whitney U Test was used to compare number of the four most commonly collected species among different trap types, and to evaluate differences among different trap types.

 All statistical tests were conducted using SPSS (version 16.0, IBM). 

Canonical Correspondence Analysis (CCA) is a multivariate method widely used to analyze the relationships between species and environmental variables [25-28]. In the present study, CCA (CCA, CANOCO version 4.5) was used to examine the relationship between the abundance of the mosquito species and the meteorological variables including monthly average temperature, precipitation, and relative humidity. In view of the high species richness and many uncommon species being collected in very small number, species whose abundance was less than 1% of the total were excluded from this analysis. We constructed two contingency tables: data on the abundance of mosquito species collected in each month of the year, and data on the three meteorological variables. CCAs then were run separately for Mangshi and Ruili, since the areas differed in meteorological characteristics. Thus, two ordinations were generated, expressing the main relationships between species and each of the climatic variables; and two ordination biplots were constructed to aid interpretation of temporal patterns observed in the selected species. 

### Virus Isolation and Identification

Mosquito pools were homogenized with minimum essential medium (MEM) in a sterile grinder and centrifuged as descibed [29-31]. To isolate the virus, 150 μL of supernatant from each pool was inoculated onto monolayers of baby hamster kidney-BHK-21, African green monkey kidney- Vero-Osaka, and *Aedes albopictus* C6/36 cells in 24-well cell culture plates. After seven days, 150-μL supernatant from the previous passage was inoculated onto a new passage in cells as above. Inoculated cells were maintained at 37 °C or 28 °C in an incubator with 5% CO_2_, and examined daily for cytopathic effect (CPE) from 1-7 days post-inoculation. A specimen was considered to be positive if it caused CPE in three successive cell passages. Infected cell supernatants were harvested and stored at -80°C until further identification.

Reverse-transcription polymerase chain reaction (RT-PCR) was employed to identify the isolates. The following primers were chosen according to the distribution of arboviruses in Southeast Asia: flavivirus-specific primers [32], alphavirus-specific primers [33], bunyavirus-specific primers [6], and primers to detect the JEV envelope gene [34], GETV E2 gene [35], VP6 gene of *Orbivirus* (Family *Reoviridae*) [18], VP7 gene of YUOV [18], partial NS1 gene of densovirus (Genus *Densovirus*, Family *Densoviridae*) [30,36], and S gene of Oya virus (OYV) (Simbu serogroup, Genus *bunyavirus*, Family *Bunyaviridae*) [31] (Table 2). Viral RNA was extracted by using the QIAamp Viral RNA Mini Kit (Qiagen, Valencia, CA), and cDNA was synthesized by using Ready-To-Go You-Prime First Strand Beads (American Amersham Pharmacia Biotech, Piscataway, NJ) according to the manufacturer’s instructions. With the above-mentioned primers in order of descending taxon from genus to species, each of the positive isolates was tested until PCR products were visible in gel electrophoresis.

**Table 2:** Primers used to identify and sequence viruses isolated from mosquitoes in this study.

**Primers**	**Primers sequence (5′ to 3′)**	**Size, base pairs**
**Alphavirus**		
M2W	YAGAGCDTTTTCGCAYSTRGCHW	
cM3W	ACATRAANKGNGTNGTRTCRAANCCDAYCC	434
M2W2	TGYCCNVTGMDNWSYVCNGARGAYCC	
GETVCaF	TGGTCCCGCATCGGATTGAG	
GETVCaR	CTTTCATGTCATGCCCGGCGATG	1450
GETVE2F	GTAACAATAGTGCACGCCACC	
GETVE2R	GGCAGCAGCAAAGCAGGTTC	1400
**Flavivirus**		
FU1	TACCACATGATGGGAAAGAGAGAGAA	
cFD2	GTGTCCCAGCCGGCGGTGTCATCAGC	310
JEPrM251F	CGTTCTTCAAGTTTACAGCATTAGC	
JEPrM743R	CGYTTGGAATGYCTRGTCCG	492
JEPrM925R	CCYRTGTTYCTGCCAAGCATCCAMCC	
JE955F	TGYTGGTCGCTCCGGCTTA	
JE2536R	AAGATGCCACTTCCACAYCTC	1581
**Bunyavirus**		
BUP	ATGACTGAGTTGGAGTTTGATGTCGC	
BDW	TGTTCCTGTTGCCAGGAAAAT	250
**Densovirus**		
DNV-3F	TGTCTCTTTCTCTTGGTATTTCTTC	
DNV-3R	CATACTACACATTCGTCCTCCAC	903
**Yunnan obivirus**		
YUOVSeg7S1	AGCATTCGGTACGCAGTATCTCG	
YUOVSeg7R1	GCCGAGCCGATCATGTCACG	453
YUOVSeg7S2	GAACGACAACGCATTTTGA	
YUOVSeg7R2	CACGTTGTTCCTGCACTTGGTCTG	369
**Orbivirus**		
6-1-2F/230F	ATGCGTATGGAGGAAGAG	
6-1-2R/1202R	AAAGTTGAGGCACCAGTA	972
**Oya virus**		
Oya-S-21f	GACGCCGAGGCCCAATGTTA	
Oya-S-334r	CAAGCGGGAAGAAGGTGAATGTC	313

Note: F means forward primer; R means reverse primer. M: C/A; W: A/T; Y: C/T; K: G/T; R: G/A; V: G/A/C; D: T/

Sequencing was performed by Beijing BioMed Biotechnology Co., Ltd. Initial sequence assembly and analysis were conducted by using the ATGC software package Version 4.0 (GENETYX Corp, Tokyo, Japan). Clustal X (Version 1.83) and MegAlign (DNASTAR, Madison, USA) were used for homology and alignment analysis, and MEGA 3.1 for phylogenetic analysis and tree construction, based on the neighbor-joining assay. The bootstrap value (the number of replications) was 1,000.

## Results

### Species Richness and Diversity of Mosquitoes

Over the course of the 12-month study, a total of 85,307 female mosquitoes comprised of 43 species representing seven genera were collected using the three types of mosquito traps in villages near the cities of Mangshi and Ruili (Table 3). Of the total number, 82% were *Culex* (15 spp.) and 16% were *Anopheles* (15 spp.). Representatives of *Aedes* (four spp.), *Armigeres* (three spp.), *Mansonia* (three spp.), *Uranotaenia* (two spp.), and *Mimomyia* (one spp.) constituted the remaining 2% of the collections.

**Table 3:** Summary of mosquito species collected in Mangshi and Ruili during Jan-Dec, 2010.

**Species**	**Ruili**					**Mangshi**				
	**TT**	**LT**	**GT**	**total**	**%**	**TT**	**LT**	**GT**	**total**	**%**
*Cx. quinquefasciatus* Say	264	207	3,410	3,881	10.47	341	211	5,646	6,198	12.84
*Cx. tritaeniorhynchus* Giles	23,997	2,865	19	26,881	72.55	29,998	1,168	106	31,272	64.81
*Cx. mimeticus* Noe	4	5	2	11	0.03	1	2	0	3	0.01
*Cx. theileri* Theobald	10	6	1	17	0.05	26	1	1	28	0.06
*Cx. bitaeniorhynchus* Giles	12	8	0	20	0.05	2	2	1	5	0.01
*Cx..halifaxi* Theobald	0	0	1	1	0.00	0	0	2	2	0.00
*Cx. pallidothrax* Theobald	9	8	15	32	0.09	0	2	7	9	0.02
*Cx. fuscanus* Wiedemann	9	18	8	35	0.09	9	16	6	31	0.06
*Cx. annulus* Theobald	96	252	1	349	0.94	165	65	1	231	0.48
*Cx. nigropunctatus* Edwards	20	39	15	74	0.20	4	4	4	12	0.02
*Cx. gelidus* Theobald	473	26	0	499	1.35	5	1	0	6	0.01
*Cx. jacksoni* Edwards	0	1	0	1	0.00	0	0	0	0	0.00
*Cx fuscocephalus* Theobald	853	113	2	968	2.61	37	0	1	38	0.08
*Cx. bailyi* Barraud	0	10	0	10	0.03	0	0	0	0	0.00
*Cx. pseudovishnui* Colless	0	1	0	1	0.00	0	1	0	1	0.00
*An. sinensis* Wiedemann	1,029	64	1	1,094	2.95	6,404	141	11	6,556	13.59
*An. maculatus* Theobald	8	0	0	8	0.02	2	1	0	3	0.01
*An. minimus* Theobald	7	2	0	9	0.02	3	2	0	5	0.01
*An. culicifacies* Giles	1	0	0	1	0.01	4	0	0	4	0.01
*An. vagus* Donitz	279	41	2	322	0.87	536	17	45	598	1.24
*An. peditaeniatus* Leicester	1,065	322	0	1,387	3.74	2,487	421	11	2,919	6.05
*An. tessellatus* Theobald	512	111	1	624	1.68	67	4	4	75	0.16
*An.barbirostris* Van der Mulp	33	2	0	35	0.09	1	2	0	3	0.01
*An. kochi* Donitz	19	4	0	23	0.06	1	0	0	1	0.00
*An. splendidus* Koidzumi	7	0	0	7	0.02	0	0	0	0	0.00
*An. annularis* Van der Mulp	1	0	0	1	0.00	0	0	0	0	0.00
*An. crawfordi* Reid	6	3	0	9	0.02	1	0	0	1	0.00
*An. aconitus* Donitz	0	1	0	1	0.00	0	0	0	0	0.00
*An.gigasbaileyi* Edwards	2	0	0	2	0.01	4	0	0	4	0.01
*Ae. elsiae* Barraud	341	26	0	367	0.99	173	25	0	198	0.41
*Ae. lineatopennis* Ludlow	3	2	0	5	0.01	0	0	0	0	0.00
*Ae. albopictus* Skuse	3	1	1	5	0.01	0	1	0	1	0.00
*Ae. annandalei* Theobald	0	2	0	2	0.01	0	0	0	0	0.00
*Ar. subalbatus* Coquillett	89	35	60	184	0.50	23	7	14	44	0.09
*Ar. inchoatus* Barraud	1	2	0	3	0.01	0	0	0	0	0.00
*Ar. omissus* Edwards	0	1	0	1	0.00	0	0	0	0	0.00
*Mm. luzonensis* Ludlow	20	13	1	34	0.09	0	1	0	1	0.00
*Ur. maxima* Leicester	1	3	0	4	0.01	0	0	0	0	0.00
*Ur. novobscura* Barraud	0	5	0	5	0.01	0	0	0	0	0.00
*Ma. uniformis* Theobald	6	2	0	8	0.02	0	0	0	0	0.00
*Ma. annulifera* Theobald	123	4	0	127	0.34	5	1	0	6	0.01
*Ma. dives* Schiner	1	0	0	1	0.00	0	0	0	0	0.00
**Totals**	**29,307**	**4,205**	**3,540**	**37,052**		**40,299**	**2,096**	**5,860**	**48,255**	
**Number of Species**	**35**	**35**	**16**	**43**		**24**	**23**	**15**	**29**	
**Simpson Diversity Index**					**0.54**					**0.46**

Notes: TT: Traditional light traps; LT: CDC light traps (with dry ice); GT: CDC gravid traps; *Cx: Culex*; *An: Anopheles; Ae*: *Aedes; Ar*: *Armigeres; Mm*: *Mimomyia; Ur*: *Uranotaenia; Ma*: *Mansonia.*

**Figure 2:**
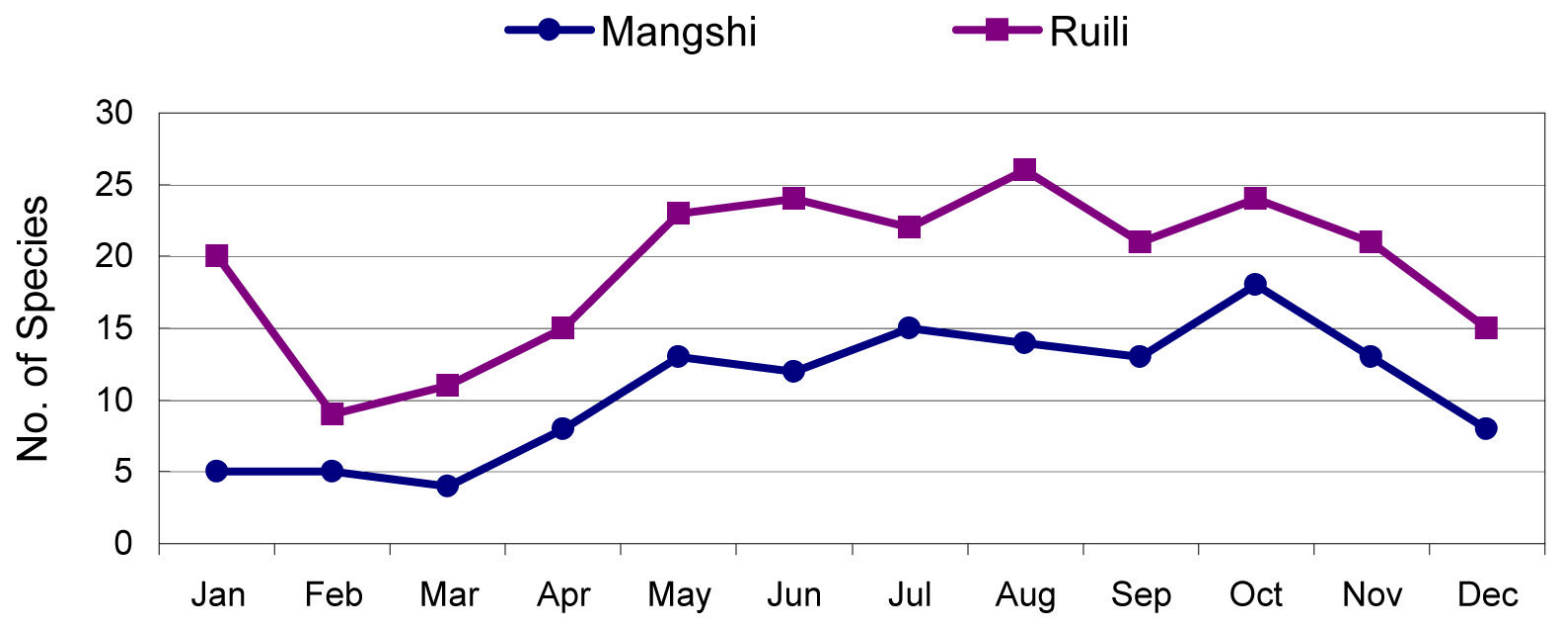
Comparison of species richness (the number of species) between Manshi and Ruili for each month , 2010.

The species richness (number of species) was significantly greater in Ruili than in Mangshi (43 and 29, respectively, *t*=4.19, *P*<0.0001) (Table S1 and Table 3). The Simpson Diversity Index of Mangshi differed significantly from that of Ruili (Ruili=0.54; Mangshi=0.46; *t* =2.66, *P*=0.014) (Table S2 and Table 3). Figure 2 shows the seasonal pattern in species richness, with more species collected in Ruili than in Mangshi during each month of the year. 

**Figure 3:**
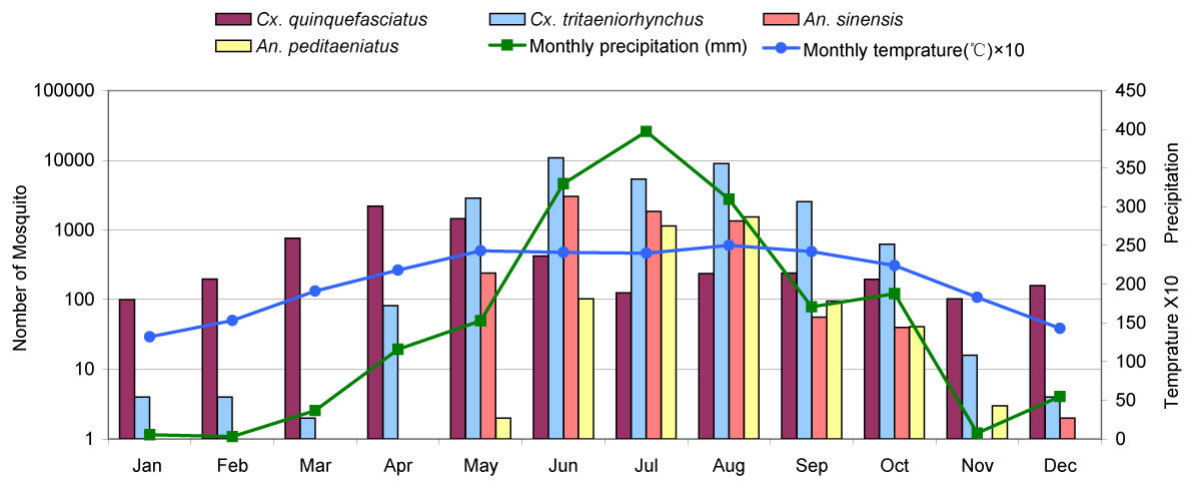
Monthly average temperature, total precipitation and abundance of the four predominant mosquito species collected in Mangshi, 2010.

**Figure 4:**
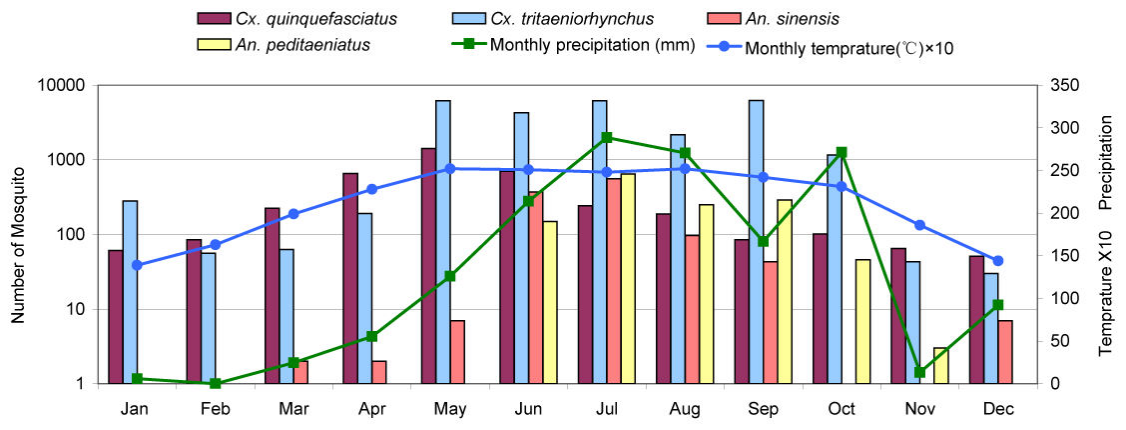
Monthly average temperature , total precipitation and abundance of the four predominant mosquito species collected in Ruili, 2010.

Species richness was generally higher during the summer and fall, with 26 species being collected during August in Ruili and 18 species collected during October in Mangshi (Table 4 and Figure 2). Only *Culex tritaeniorhynchus* Giles and *Culex quinquefasciatus* Say were collected in moderate to high numbers every month of the year (Figures 3 and 4, and Figure S1).

**Table 4:** Species richness (number of mosquito species) each month during 2010 in Mangshi and Ruili.

**City**	**Species Richness (Number of species)**											
	Jan	Feb	Mar	Apr	May	Jun	Jul	Aug	Sep	Oct	Nov	Dec
Mangshi	5	5	4	8	13	12	15	14	13	18	13	8
Ruili	20	9	11	15	23	24	22	26	21	24	21	15

In both sampling locations, *Cx. tritaeniorhynchus* was by far the most frequently collected species, comprising 72.5% of the total in Ruili and 64.8% in Mangshi. In Ruili, this was followed by *Cx. quinquefasciatus* (10.5% of total), *Anopheles peditaeniatus* Leicester (3.7%) and *Anopheles sinensis* Wiedamann (2.9%), In Mangshi, *An. sinensis* was the second-most abundant species (13.5% of total) followed by *Cx. quinquefasciatus* (12.8%) and *An. peditaeniatus* (6.0%). These four species were referred to as the common species. 

The total number of female mosquitoes collected was significantly greater in Mangshi (n=48,255) than in Ruili (n=37,025) (Z=38.36, *P*<0.00001). The number collected for each of the common species was significant higher in Manhsi than in Ruili (Z= 23.08, 18.21, 60.68, and 29.37, respectively for *Cx. tritaeniorhynchus, Cx. quinquefasciatus, An. sinensis* and *An. peditaeniatus*, *P*<0.00001) (Table S3). 

### Seasonal Abundance of Mosquitoes, Temperature, Precipitation and Humidity

**Figure 5:**
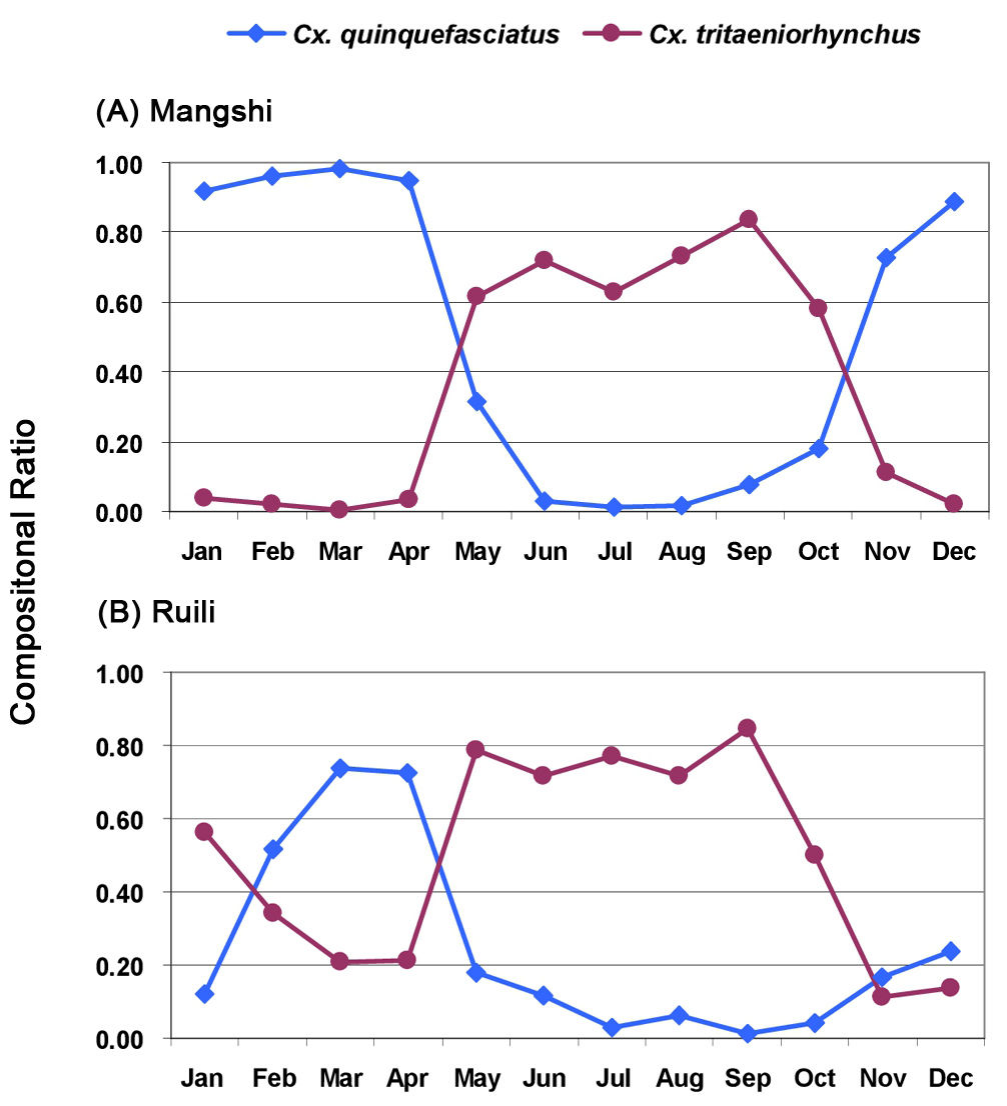
Compositional changes of *Cx. quinquefasciatus* and *Cx. tritaeniorhynchus* in total mosquitoes collected in Mangshi and Ruili, 2010.

Mosquitoes were collected during each month of the year in both cities of Mangshi and Ruili. Abundance of the four most common mosquito species, temperature, and rainfall patterns occurring in Mangshi and Ruili are shown in Figures 3 and 4, respectively. Rainfall and temperature patterns were similar in both sampling sites. January through March were relatively dry, rainfall increased each month until July, stayed relatively high through the rainy season until September-October, then declined during November and December. Average monthly temperatures were at their minimum (approx 15 °C) in January and climbed gradually to approximately 24 °C in May and stayed at approximately that level through September, then gradually declined during October through December. In Mangshi (Figure 3), *Cx. quinquefasciatus* was the most abundant species collected from January through April. Subsequently *Cx. tritaeniorhynchus* abundance exceeded that of *Cx. quinquefasciatus* and remained that way until November (Figure 5 and Figure S1). This seasonal change in abundance of these two species was also observed in Ruili, where *An. sinensis* and *An. pediateniatus* were not collected until May. Their abundance increased through the course of the rainy-hot season and declined as temperatures and rainfall decreased in September through December. The temperature and precipitation patterns in Ruili were similar (Figure 4), however *An. sinensis* were first collected in March (three months earlier than observed in Mangshi), increased gradually until the population peaked in June and July, and then gradually decreased until December. The pattern of *An. peditaeniatus* in Ruili was similar to that seen in Mangshi, with this species first being collected during June and persisting until November. 

Temporal trends in the abundance of each species were also illustrated by the ordination bioplots constructed by CCA. In both Manshi and Ruili (Figures S2 and S3), *Cx. tritaeniorhynchus*, *An. sinensis* and *An. pediateniatus*, as well as *Anopheles vagus* Donitz in Mangshi, displayed considerable associations with the three meteorological variables, whereas *Cx. quinquefasciatus* showed the poorest relationship with temperature, humidity and rainfall. Meanwhile, for the four common species collected from either of sampling sites, the three meteorological factors, which appeared to exert similar effect upon each species, tended to be identified as one “common factor”, largely due to their co-variation in seasonal change. 

In Ruili (Figure S3), *Culex fuscocephalus* Theobald, *Culex gelidus* Theobald, and *Anopheles tessellatus* Theobald did not have strong associations with temperature. The two *Cx*. spp. were trapped in greatest abundance in October, while the latter *An. tessellatus* sustained its peak from June through October. These results suggested that local mosquito species, even those belonging to the same genus, did not display the same temporal trend in seasonal abundance variation. 

### Comparison of Trap Types

By using different trap types at the same locations, we were able to compare their ability to sample the local mosquito populations. In both Ruili and Mangshi sampling sites, the TT collected significantly more host seeking mosquitoes than the LT (Z=229.7, *P*<0.0001) and GT (Z=214.2, *P*<0.0001) (Table 3). In Ruili and Mangshi respectively, the TT collected approximately 7X and 19X more total mosquitoes than the LT, primarily resulting from the very large numbers of *Cx. tritaeniorhynchus* collected by the TT. However, both the TT and LT collections indicated similar species richness in the two sites; despite collecting many fewer total specimens, the LT collected 35 species in Ruili and 23 in Mangshi, compared to 35 and 24 by the TT in Ruili and Mangshi, respectively. The GT collected significantly more *Cx. quinquefasciatus* than the TT (Mann-Whitney U=42, *P*=0.0001) or LT (Mann-Whitney U=27.5, *P*=0.0001) (Figure S4) with differences ranging from 12X to 16X the number of *Cx. quinquefasciatus* than the TT, and from 16X to 27X the number of *Cx. quinquefasciatus* than the LT in Ruli and Mangshi, respectively. GT (n=15 in Mangshi; n=16 in Ruili) collected fewer species than the TT (n=24 in Mangshi; n=35 in Ruili) or LT (n=23 in Mangshi; n=35 in Ruili) (*P*<0.05) (Table 3). In addition, TT collected significantly more *An. sinensis* and *An. peditaeniatus* than LT (Mann-Whitney U=135.0, *P*=0.0001) or GT (Mann-Whitney U=83.5, *P*=0.0001) (Figure S4).

### Virus Isolation and Identification

Of the 85,307 mosquitoes collected in Ruili and Mangshi during 2010, 69,209 specimens were tested for virus isolation in 1,430 pools (Table 5). From these, 26 virus isolates were obtained (Table 6). Of these, 13 strains were identified as JEV, five of which were from mosquitoes collected in Ruili (four from *Cx. tritaeniorhynchus* and one from *Culex pallidothorax* Theobald) and eight were from mosquitoes collected in Mangshi (seven from *Cx. tritaeniorhynchus* and one from *Cx. gelidus*). Four strains of GETV were isolated from mosquitoes collected in Ruili, one isolate from each of *Cx. tritaeriorhynchus*, *An. sinensis*, *Cx. fuscocephalus* and *Culex annulus* Theobald. Seven strains of *Culex pipiens pallens* densovirus (CppDNV) (Genus *Densovirus*, Family *Densoviridae*) were isolated; three from mosquitoes collected in Ruili (one from each of *Cx. quinquefasciatus, Cx. tritaeriorhynchus*, and *Armigeres subalbatus* Coquillett) and four from mosquitoes collected in Mangshi ( three from *An. sinensis* and one from *Cx. quinquefasciatus*). One strain of OYV was isolated from *Cx. quinquefasciatus* and one strain of *Orbivirus genus* (unidentified into species) was isolated from *An. sinensis* collected in Ruili. 

**Table 5:** Summary of mosquito species collected and tested for virus in Mangshi and Ruili during Jan-Dec, 2010.

**Mosquito species**	**Mangshi**			**Ruili**		
	**No.collected**	**No. tested**	**pools**	**No. collected**	**No. tested**	**pools**
*Cx. quinquefasciatus*	6,198	6,198	113	3,881	3,881	82
*Cx. tritaeniorhynchus*	31,272	21,772	248	26,881	21,702	233
*Cx. mimeticus*	3	3	3	11	11	9
*Cx. theileri*	28	28	13	17	17	8
*Cx. bitaeniorhynchus*	5	5	4	20	20	12
*Cx. .halifaxi*	2	2	2	1	1	1
*Cx. pallidothrax*	9	9	4	32	32	20
*Cx. fuscanus*	31	31	17	35	35	19
*Cx. annulus*	231	231	14	349	349	18
*Cx. nigropunctatus*	12	12	6	74	74	21
*Cx. gelidus*	6	6	3	499	499	27
*Cx. jacksoni*				1	1	1
*Cx. fuscocephala*	38	38	11	968	968	42
*Cx. bailyi*				10	10	3
*Cx. pseudovishnui*	1	1	1	1	1	1
*An. sinensis*	6,556	5,396	71	1,094	1,094	35
*An. maculatus*	3	3	3	8	8	5
*An. minimus*	5	5	4	9	9	6
*An. culicifacies*	4	4	3	2	2	2
*An. vagas*	598	598	20	322	322	22
*An. peditaeniatus*	2,919	2,660	40	1,387	1,387	28
*An. tessellatus*	75	75	15	624	624	35
*An. barbirostris*	3	3	3	35	35	18
*An. kochi*	1	1	1	23	23	10
*An. splendidus*				7	7	4
*An. annularis*				1	1	1
*An. crawfordi*	1	1	1	9	9	6
*An. aconitus*				1	1	1
*An.gigas baileyi*	4	4	3	2	2	2
*Ae. elsiae*	198	198	22	367	367	29
*Ae. lineatopennis*				5	5	4
*Ae. albopictus*	1	1	1	5	5	5
*Ae. annandalei*				2	2	2
*Ar. subalbatus*	44	44	14	184	184	29
*Ar. inchoatus*				3	3	3
*Ar. omissus*				1	1	1
*Mm. luzonensis*	1	1	1	34	34	17
*Ur. maxima*				4	4	4
*Ur. novobscura*				5	5	3
*Ma. uniformis*				8	8	5
*Ma. Annulifera*	6	6	1	127	127	11
*Ma. dives*				1	1	1
**total**	**48,255**	**37,336**	**642**	**37,052**	**31,873**	**788**

**Table 6:** Summary of viruses isolated from mosquitoes collected in Ruili and Mangshi, Yunnan Province 2010.

**No.**	**Isolate identifier**	**Species of mosquito**	**Collection date**	**Trap type**	**Sites**	**Virus**
1	DH10M107	*Cx. quinquefasciatus*	March 16, 2010	GT	Mangshi	CppDNV
2	DH10M390	*Cx. tritaeriorhynchus*	May 14, 2010	TT	Ruili	GETV
3	DH10M397	*Cx. quinquefasciatus*	May 13-14, 2110	TT, LT and GT	Ruili	CppDNV
4	DH10M429	*An. sinensis*	June 16-17, 2010	TT, LT and GT	Mangshi	CppDNV
5	DH10M500	*An. sinensis*	June 16, 2010	TT	Mangshi	CppDNV
6	DH10M530	*An. sinensis*	June 16, 2010	TT	Mangshi	CppDNV
7	DH10M585	*Cx. tritaeriorhynchus*	June 15, 2010	TT	Ruili	JEV
8	DH10M609	*Cx. tritaeriorhynchus*	June 15, 2010	TT	Ruili	JEV
9	DH10M619	*Cx. tritaeriorhynchus*	June 14, 2010	TT and LT	Ruili	CppDNV
10	DH10M635	*Cx. tritaeriorhynchus*	June 15, 2010	TT	Ruili	JEV
11	DH10M648	*Cx. tritaeriorhynchus*	July 14-15, 2010	TT and LT	Mangshi	JEV
12	DH10M662	*Cx. gelidus*	July 15, 2010	LT	Mangshi	JEV
13	DH10M663	*Cx. tritaeriorhynchus*	July 15, 2010	TT	Mangshi	JEV
14	DH10M701	*Cx. tritaeriorhynchus*	July 15, 2010	TT	Mangshi	JEV
15	DH10M742	*Cx. tritaeriorhynchus*	July 15, 2010	TT	Mangshi	JEV
16	DH10M865	*Cx. tritaeriorhynchus*	August 17, 2010	TT	Mangshi	JEV
17	DH10M872	*Cx. tritaeriorhynchus*	August 16, 2010	TT and LT	Mangshi	JEV
18	DH10M919	*Cx. tritaeriorhynchus*	August 17, 2010	TT	Mangshi	JEV
19	DH10M978	*Cx. tritaeriorhynchus*	August 15, 2010	TT	Ruili	JEV
20	DH10M984	*Cx. pallidothrax*	August 14, 2010	GT	Ruili	JEV
21	DH10M1013	*Cx. quinquefasciatus*	August 14-15, 2010	TT and GT	Ruili	OYV
22	DH10M1019	*An. sinensis*	August 14-15, 2010	TT	Ruili	orbivirus
23	DH10M1102	*Cx. fuscocephala*	September 15-16, 2010	TT	Ruili	GETV
24	DH10M1105	*An. sinensis*	September 15-16, 2010	TT	Ruili	GETV
25	DH10M1106	*Cx. annulus*	September 15-16, 2010	TT, LT and GT	Ruili	GETV
26	DH10M1170	*Ar. subalbatus*	September 15-16, 2010	TT, LT and GT	Ruili	CppDNV

All 26 strains of viruses were isolated from the mosquitoes collected from March through September, with JEV in June, July and August; GETV in May and September; OYV and Orbivirus in August; CppDNV in March, May, July and September (Table 6).

We calculated the minimum infection rate (MIR) of JEV in *Cx. tritaeniorhynchus* populations in Mangshi and Ruili during June, July and August, the months during which JEV was detected in mosquitoes (Table 7). In Ruili, the MIR ranged from 0 to 0.7/1000 and in Mangshi the MIR ranged from 0 to 0.9/1000 mosquitoes.

**Table 7:** MIR by month of JEV in *Culex tritaeniorhynchus* collected in Ruili and Mangshi, Jun-Aug, 2010.

	**June**	**July**	**August**
**Ruili**			
No. of specimens	4,281	5,179	2,171
No. of positive pools	3	0	1
MIR	0.70	0	0.46
**Mangshi**			
No. of specimens	6,784	4,387	4,465
No. of positive pools	0	4	3
MIR	0	0.91	0.67

Note: Minimum Infection Rate expressed as number infected/1000 tested.

## Discussion

Over a 12-month period in 2010, mosquito collections using 3 distinct traps demonstrated that mosquitoes are active year round and documented significantly more species diversity, 29 species from six genera in Mangshi and 43 species from seven genera in Ruili than the 25 species previously reported from surveys limited to July through August in this part of China [37-40].

Four mosquito species accounted for the majority of the mosquitoes collected in both areas. *Cx. tritaeniorhynchus* was, by far, the most abundant mosquito, consistent with previous studies in 15 counties near the Yunnan Province border with Myanmar, Laos and Vietnam [19,41]. Like *Cx. tritaeniorhynchus*, *An. sinensis* and *An. pediateniatus* were also abundant in the summer months. However, in previous studies [19-21], *An. sinensis* was reported as a very common species, whereas *An. pediateniatus was* collected only in very low numbers. In our study another species, *Cx. quinquefasciatus* was identified as one of the predominant species in the area, and was shown to be active at moderate to high levels throughout the year, contrary to reports in previous studies. This finding is due to the inclusion of the GT in the sampling strategy. The GT accounted for 87.9% and 91.1% of *Cx. quinquefasciatus* collected in Mangshi and Ruili, respectively. In previous studies only using the TT, no *Cx. quinquefasciatus* were collected in Mangshi [37] and very few were collected in Ruili [38] and numerous other sites in the region [19,39,40]. Our study also supported observations that UV light (TT) or incandescent light and CO_2_ (LT) were virtually unattractive to host-seeking *Cx. quinquefasciatus*.

The greater species richness found in Ruili (43 species) compared to Mangshi (29 species) is mainly the result of *Culex, Anopheles, Aedes, Armigeres, Uranotaenia*, and *Mansonia* species in Ruili. As a rule, species diversity is proportional to altitude, but not to the number of specimens collected [42]. Lower altitude and higher temperature, rainfall and humidity, together with the more southerly geographic location (Table 1) may result in Ruili supporting appropriate conditions for more diverse mosquito species breeding than Mangshi. Conversely, the greater abundance of the four most common species in Mangshi suggests that more habitat for these species exists in Mangshi than in Ruili However, these results may be explained by other environmental factors associated with the different sampling sites, natural and artificial habitat structures, land use patterns, and host availability. These environmental factors are the subject of future research projects.

That mosquito breeding depends largely on appropriate climatic conditions is reflected in the enormous difference in mosquito numbers obtained during winter and summer. However, as Figures 3, 4 (and Figure S1) indicated, the *Cx. quinquefasciatus* population peaked in April and May, respectively for Mangshi and Ruili, and then began to decrease, while three other most abundant species attained their peaks two-three months later, or maintained this peak value for longer period (e.g. *Cx. tritaeniorhynchus*, Ruili). This may be explained by the interaction of the properties inherent in GT (i.e., attractiveness to *Cx. quinquefasciatus*) and the environmental factors associated with seasonal changes and human farming activity. At the time of year when the rainy season is approaching, temperatures are increasing and paddy fields have not been irrigated, GT appears to be a quite attractive device to trap *Cx. quinquefasciatus*. Once the paddy fields around human residences are filled with water during the rainy season, the GT, a device designed primarily to attract ovipositing gravid *Cx. quinquefasciatus*, may be outcompeted by other numerous oviposition sites. The ubiquity of flooded paddy fields during the wet growing season supports other mosquitoes, such as *Cx. tritaeniorhynchus*, which are collected in large numbers in the TT. As a consequence of the mid-April rice planting, our collecting indicates that the proportion of *Cx. quinquefasciatus* relative to *Cx. tritaeniorhynchus* decreases sharply in both cities (Figure 5 and Figure S1). 

The results demonstrate that the TT, LT and GT, each have advantages in collecting different mosquito species. The TT was the optimal mosquito trap for collecting large numbers of *Cx. tritaeriorhynchus*, *An. sinensis*, and *An. peditaeniatus*. The diversity of mosquitoes sampled collected by the LT was similar to that sampled by TT, however, the numbers collected by the LT were markedly less than those collected by TT. This is likely related to the relatively weak light source of LT, despite the presence of CO_2_ bait. The disadvantage of the TT is that it requires 220V power and is limited to use around residential areas where power is available. LT can be used in remote locations and to sample habitats where electrical outlets are not available. The GT was by far the optimal mosquito trap for *Cx. quinquefasciatus*. 

We used three trap types to better characterize the structure of local mosquito communities, resulting in new indications that *Cx. quinquefasciatus* and *An. pediateniatus* were abundant in the area, Our study represents, to our knowledge, the first time GT have been used in China, and we recommend that it be incorporated into mosquito surveillance programs to provide a more thorough and complete insight to the mosquito community structure wherever surveillance programs are conducted. Only when more sampling tools with distinct and well-defined properties are applied in mosquito collecting, can we better define mosquito community structure in the field.

Knowledge of the year-round activity of *Cx. quinquefasciatus* in this area is important since this species is not only the well-known vector transmitting JEV [20,43], but one of the most important vectors of West Nile virus (WNV) [44,45]. Though not previously found in Yunnan Province, WNV global distribution has been expanding and WNV and other zoonotic flaviviruses have been found in southeast Asia [46,47] and could establish transmission foci in areas where competent vectors like *Cx. quinquefasciatus* exist.

The western borderland of Yunnan Province is a known epidemic area for JE [1,48], so detecting JEV in mosquito species that have been previously associated with JEV transmission was not surprising [2,3,8,19,20,41]. The 13 strains of JEV isolated in the present study were all determined to be genotype 1, which is consistent with the recent isolation of genotype 1 JEV in the cerebrospinal fluid samples from JE patients in this area [49]. This is in contrast to the JEV isolated in the same area in the 1980’s which were all genotype 3 [9,34]. These observations suggest that genotype 1 has replaced genotype 3 and has become the prevalent genotype of JEV in the area, consistent with other results demonstrating the emergence of genotype 1 JEV as the dominant genotype found in Asia [50,51]. Some authors proposed that genotype 1 JEV originated in Southeast Asia [52-54]. In that case, this genotype was likely introduced into Yunnan Province and other areas of China in the past decade. However, many explanations as to its transmission pattern and mechanism in Asia remained speculative. A total of 80 human JE cases were reported during 2010 through 2012 in our study area, which occurred between May (two cases) and November (one), and peaked in July (34) [48]. In our study, JEVs were isolated in June through August, consistent with the peak of human cases. Although human cases were not reported in December through April of the next year, and no JEV was isolated other than June-August in the present study, the year-round activity of *Cx. tritaeriorhynchus* and *Cx. quinquefasciatus* was confirmed (Figure 3 and 4), implying that JEV could circulate in local mosquitoes all year round.

GETV was first isolated from *Cx. gelidus* in Malaysia [55], and is widely distributed in Southeast Asia and east Asia. GETV is pathogenic in horses, pigs, and other domestic animals [56,57], but has not been associated with disease in humans. GETV has been previously isolated from *An. sinensis* and *Ar. subalbatus* in northwest areas of Yunnan Province [6].The current results represent the first association of GETV with *Cx. tritaeriorhynchus*, *Cx. fuscocephalus* and *Cx. annulus* in Yunnan Province and indicates that *Culex* species in the China-Myanmar border area may be important GETV vectors. We have detected GETV antibody by hemagglutination inhibition test in human serum samples in areas near the Lancang River in the middle and south areas of Yunnan Province [58], suggesting human infection and a wide distribution in Yunnan Province. 

Previously, CppDNV has been isolated in China [30,36]. The seven strains of CppDNV isolated in the present study were from *An. sinensis*, *Cx. quinquefasciatus*, *Cx. tritaeriorhynchus* and *Ar. subalbatus*, indicating that CppDNV can infect many mosquito species in different genera, but this virus seems to exist only as a mosquito-carried virus, without causing human or animal infection. The orbivirus isolated from *An. sinensis* in the present study caused CPE in BHK21 cells, while the YNOV previously isolated from the south area of Yunnan Province produced CPE only in C6/36 cells [4,18]. The biological characteristics of these orbivirus isolates are apparently different and need further investigation. In the 1980s, orbiviruses were isolated in pigs and cattle [59,60], indicating that it can infect these animals, but whether the strains of orbivirus obtained in this study were pathogenic to humans and other animals remains unclear. One strain of OYV isolated in the present study was from *Cx. quinquefasciatus*. OYV, commonly infects pigs [61] and has been isolated from other mosquito species collected in northern Vietnam [62]. This is the first demonstrated association of OYV with *Cx. quinquefasciatus*. There are no reports about this virus in China. No human cases caused by this virus have been reported. Further investigations of OYV transmission dynamics should be carried out in areas where this virus has been isolated.

In the past, mosquito-borne viruses were isolated only in July and August. In the current study, the time of arboviruses isolation spanned from March through September, illustrating more detailed data on the seasonal pattern of virus cycling. Failure to recover viruses in the rest of months may be related to low number of mosquitoes that had been sampled in the relative cold season. However, the presence of some mosquito species in winter suggested that the adult mosquitoes could carry the associated viruses throughout the entire year. This information will provide a better understanding of the ecology of vector and other mosquitoes in this region, and improve understanding of factors that may influence tran-seasonal virus maintenance. In addition data derived from comprehensive, year-long studies such as this will be useful as a baseline to monitor the effects of changes in land use and climate on vector abundance and virus transmission.

For local public health agencies, our study also presented the fact that well-timed interventions to reduce human cases associated with JEV and other mosquito-borne pathogens should be carried out in April, with efforts focusing upon larval mosquito control during the period when the number of larval habitats is still low. In June and July, vector control should emphasize reducing adult mosquitoes. The abundance and the diversity of mosquitoes highlight the possibility that some exotic viruses, such as WNV, may establish in this area; routine monitoring therefore is needed. 

## Supporting Information

Figure S1 **Seasonal peaks of the four most common mosquito species collected in Mangshi and Ruili cities, Yunnan Province, 2010.**
(TIF)

Figure S2 **Mangshi CCA biplot: locations of species (>1%) relative to the first two axes derived in CCA.** Species are showed as triangles. The abbreviations represented *Cx. tritaeniorhynchus*, *Cx. quinquefasciatus*, *An. sinensis*, *An. peditaeniatus*, and *An. vagas*. Each vector represents a given meteorological variable with its mean lying at the origin. Above average values lie alone their corresponding vectors, and below average values project in opposite direction of the vector.
(TIF)

Figure S3 **Ruili CCA biplots: locations of species (>1%) relative to the first two axes derived in CCA.** Species are showed as triangles. The abbreviations represent *Cx. tritaeniorhynchus*, *Cx. quinquefasciatus*, *An. sinensis*, *An. peditaeniatus*, *Cx. fuscocephalus*, *Cx. gelidus*, and *An. tessellates*. Each vector represents a given meteorological variable with its mean lying at the origin. Above average values lie alone their corresponding vectors, and below average values project in opposite direction of the vector.
(TIF)

Figure S4 **Comparison of sampling ability of different trap types by using Mann-Whitney U test.**
(TIF)

Table S1 **The results of t-test for comparing species richness between Mangshi and Ruili.**
(DOC)

Table S2 **The results of t-test for comparing SDI between Mangshi and Ruili.**
(DOC)

Table S3 **The results of Z-test for comparing the collected number of each common species between Mangshi and Ruili.**
(DOC)
