# Supplementary figures and images for: Mosquitoes of Western Yunnan Province, China: Seasonal Abundance, Diversity, and Arbovirus Associations
Source: PLoS One. 2013 Oct 11;8(10):e77017. doi: 10.1371/journal.pone.0077017 (PMC3795637; doi:10.1371/journal.pone.0077017)

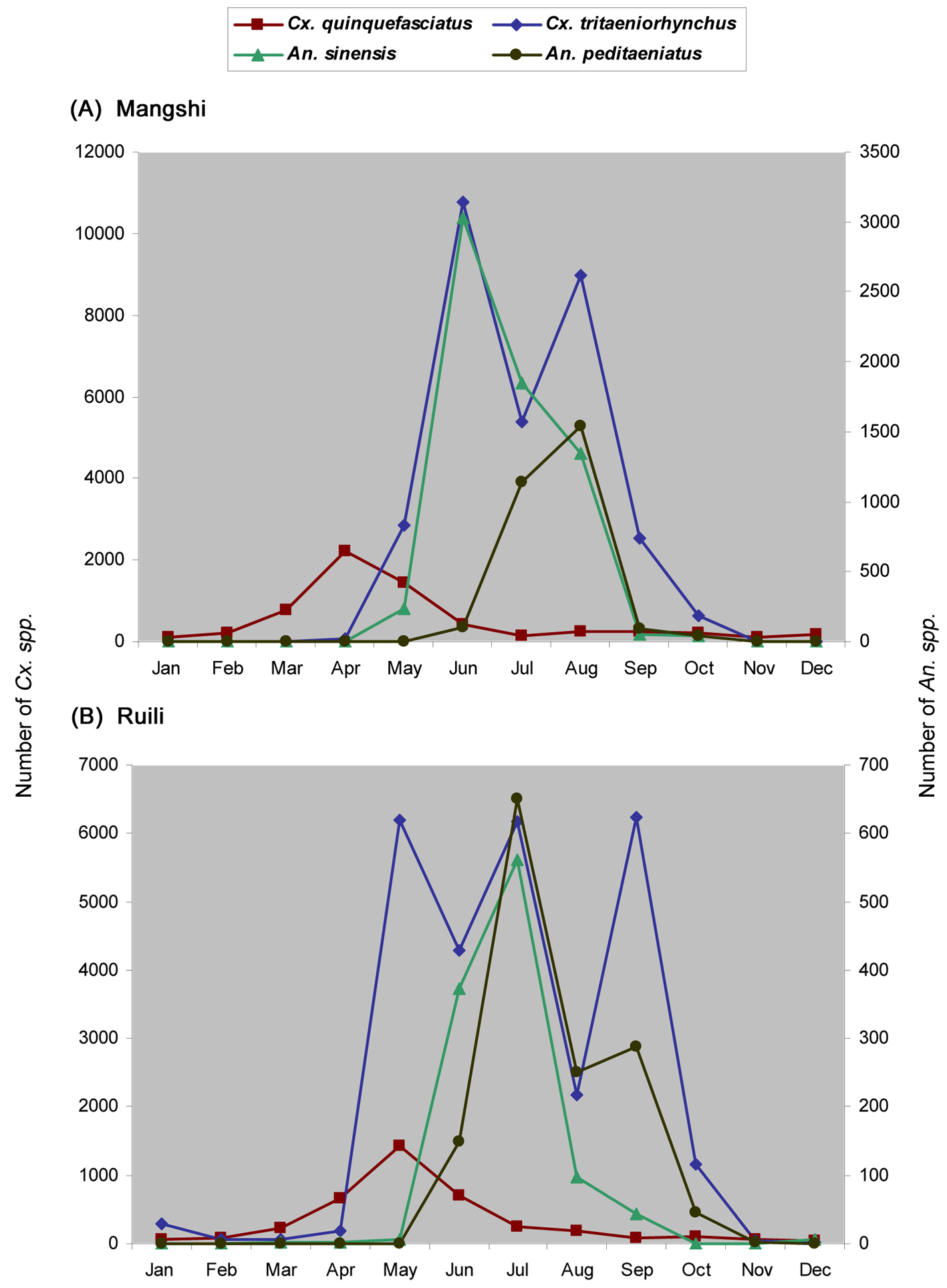

Supplement: Figure S1 — Seasonal peaks of the four most common mosquito species collected in Mangshi and Ruili cities, Yunnan Province, 2010. (TIF) [file pone.0077017.s001.tif]

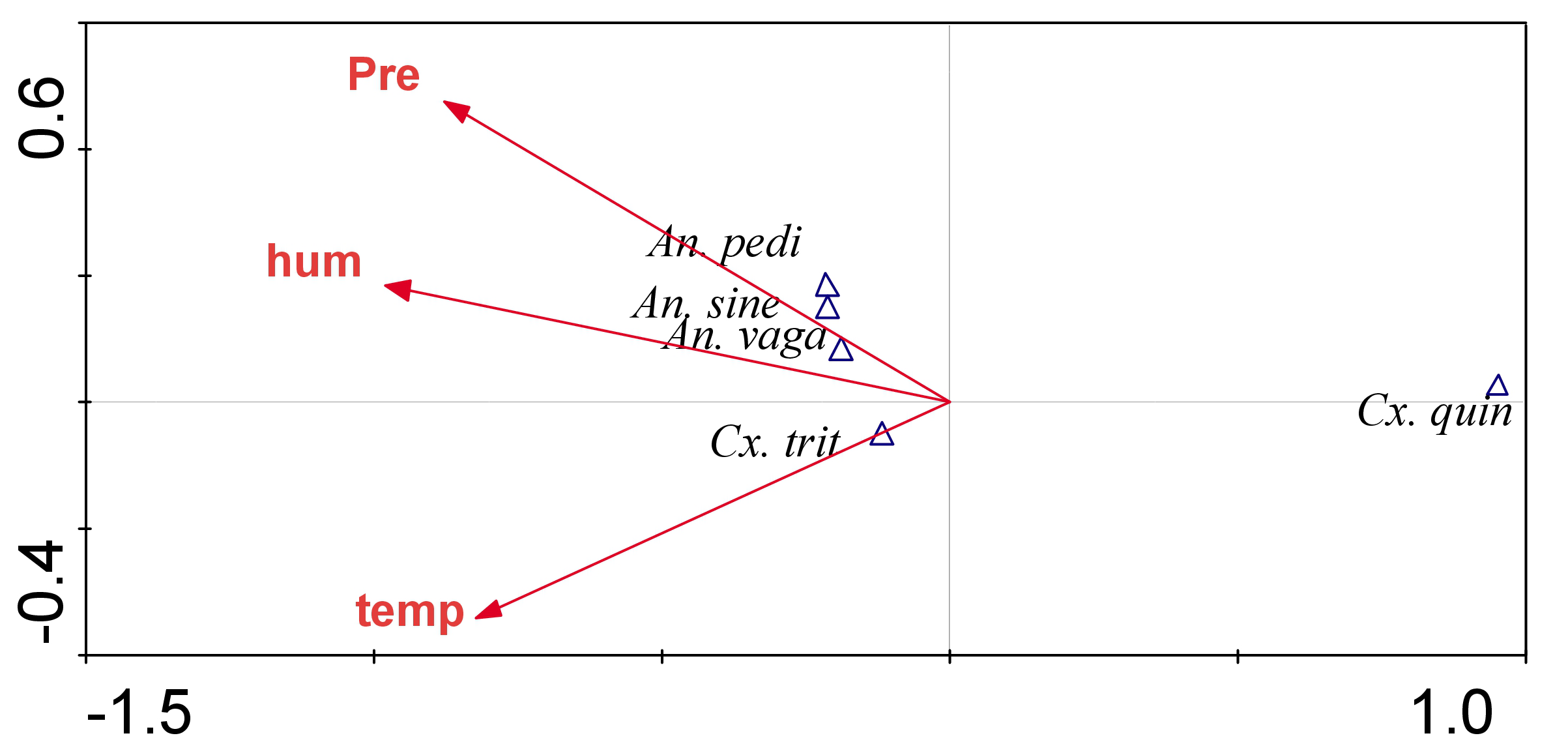

Supplement: Figure S2 — Mangshi CCA biplot: locations of species (>1%) relative to the first two axes derived in CCA. Species are showed as triangles. The abbreviations represented Cx. tritaeniorhynchus, Cx. quinquefasciatus, An. sinensis, An. peditaeniatus, and An. vagas. Each vector represents a given meteorological variable with its mean lying at the origin. Above average values lie alone their corresponding vectors, and below average values project in opposite direction of the vector. (TIF) [file pone.0077017.s002.tif]

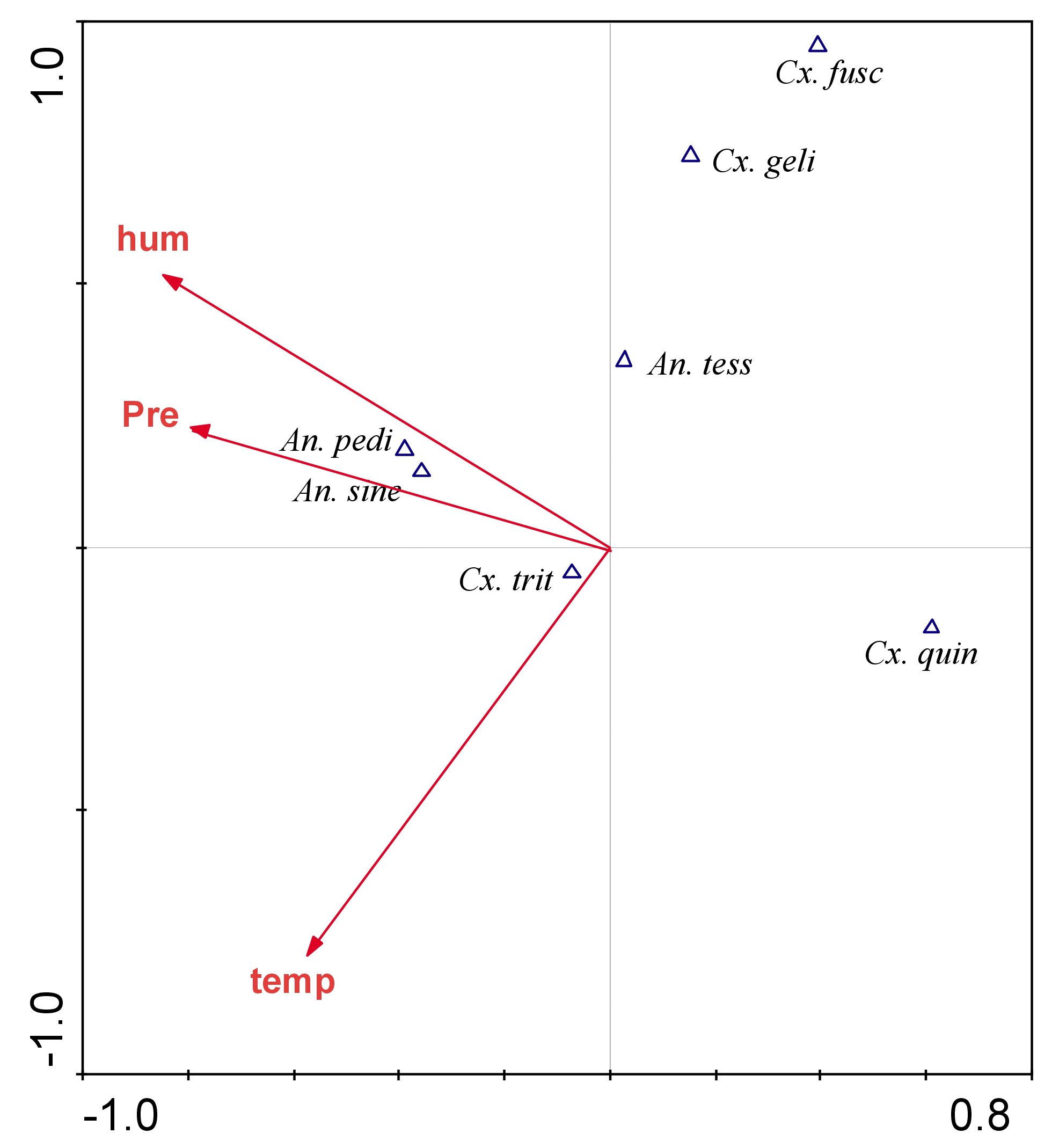

Supplement: Figure S3 — Ruili CCA biplots: locations of species (>1%) relative to the first two axes derived in CCA. Species are showed as triangles. The abbreviations represent Cx. tritaeniorhynchus, Cx. quinquefasciatus, An. sinensis, An. peditaeniatus, Cx. fuscocephalus, Cx. gelidus, and An. tessellates. Each vector represents a given meteorological variable with its mean lying at the origin. Above average values lie alone their corresponding vectors, and below average values project in opposite direction of the vector. (TIF) [file pone.0077017.s003.tif]

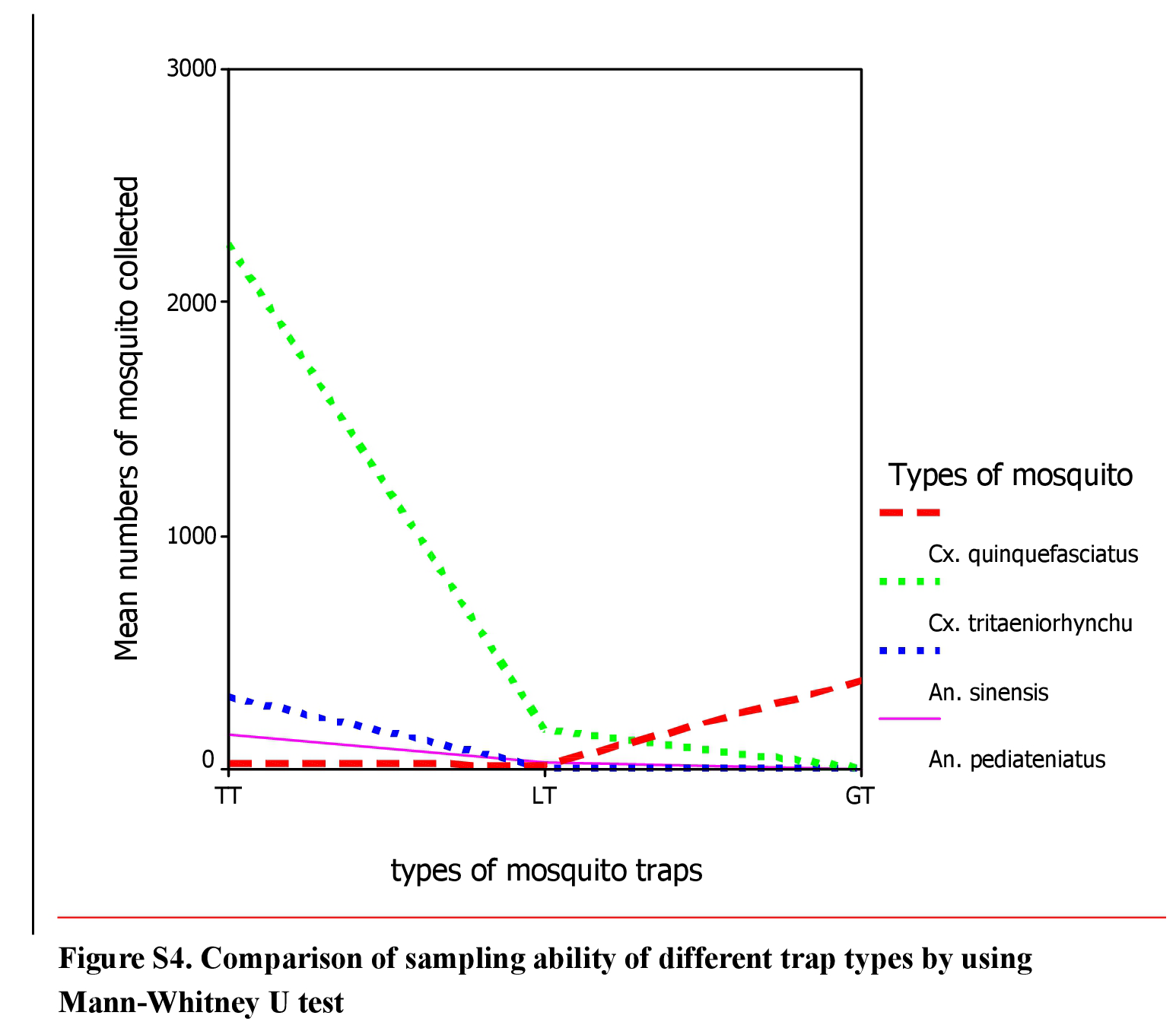

Supplement: Figure S4 — Comparison of sampling ability of different trap types by using Mann-Whitney U test. (TIF) [file pone.0077017.s004.tif]
