# Supplementary material for: Mosquitoes of Western Yunnan Province, China: Seasonal Abundance, Diversity, and Arbovirus Associations
Source: PLoS One. 2013 Oct 11;8(10):e77017. doi: 10.1371/journal.pone.0077017 (PMC3795637; doi:10.1371/journal.pone.0077017)
Supplement: Table S1 — The results of t-test for comparing species richness between Mangshi and Ruili. (DOC) [file pone.0077017.s005.doc]

**Table S1.** The results of t-test for comparing species richness between Mangshi and Ruili

| **Sites** | $\overline{x}\pm s$ | **Species no.** | **Value** |
| --- | --- | --- | --- |
| Ruili（n=12） | 19.25±5.46 | 43 | t =4.19 |
| Mangshi（n=12） | 10.67±4.52 | 29 | *P*<0.0001 |
