# Supplementary material for: Mosquitoes of Western Yunnan Province, China: Seasonal Abundance, Diversity, and Arbovirus Associations
Source: PLoS One. 2013 Oct 11;8(10):e77017. doi: 10.1371/journal.pone.0077017 (PMC3795637; doi:10.1371/journal.pone.0077017)
Supplement: Table S2 — The results of t-test for comparing SDI between Mangshi and Ruili. (DOC) [file pone.0077017.s006.doc]

**Table S2.** The results of t-test for comparing SDI between Mangshi and Ruili

| **Sites** | $\overline{x}\pm s$ | **SDI** | **Value** |
| --- | --- | --- | --- |
| Ruili（n=12） | 0.52±0.17 | 0.54 | t =2.66 |
| Mangshi（n=12） | 0.32±0.20 | 0.46 | *P*=0.014 |
