# Supplementary material for: Mosquitoes of Western Yunnan Province, China: Seasonal Abundance, Diversity, and Arbovirus Associations
Source: PLoS One. 2013 Oct 11;8(10):e77017. doi: 10.1371/journal.pone.0077017 (PMC3795637; doi:10.1371/journal.pone.0077017)
Supplement: Table S3 — The results of Z-test for comparing the collected number of each common species between Mangshi and Ruili. (DOC) [file pone.0077017.s007.doc]

**Table S3.** The results of Z-test for comparing the collected number of each common species between Mangshi and Ruili

|  | ***Cx. quinquefasciatus*** | ***Cx. tritaeniorhynchus*** | ***An. sinensis*** | ***An. peditaeniatus*** | **Total** |
| --- | --- | --- | --- | --- | --- |
| Mangshi | 6,198 | 31,272 | 6,556 | 2,919 | 48,255 |
| Ruili | 3,881 | 26,881 | 1,029 | 1,065 | 37,052 |
| Z-value | 23.08 | 18.21 | 60.68 | 29.37 | 38.36 |
| P-value | <0.00001 | <0.00001 | <0.00001 | <0.00001 | <0.00001 |
